# Inpatient burden of respiratory syncytial virus in children ≤2 years of age in Germany: A retrospective analysis of nationwide hospitalization data, 2019–2022

**DOI:** 10.1111/irv.13211

**Published:** 2023-11-24

**Authors:** Moritz Wick, Anahita Poshtiban, Rolf Kramer, Mathieu Bangert, Matthias Lange, Martin Wetzke, Oliver Damm

**Affiliations:** ^1^ Sanofi‐Aventis Deutschland GmbH Berlin Germany; ^2^ Department of Pediatric Pneumology and Allergology Universitätsklinik für Kinder‐ und Jugendmedizin Oldenburg Oldenburg Germany; ^3^ Department of Pediatric Pneumology, Allergology and Neonatology Hannover Medical School Hannover Germany; ^4^ Biomedical Research in End‐Stage and Obstructive Lung Disease Hannover (BREATH) Hannover Germany

**Keywords:** epidemiology, Germany, hospitalization, resource use and costs, respiratory syncytial virus, severe acute respiratory infections

## Abstract

**Background:**

Respiratory syncytial virus (RSV) causes respiratory tract disease in seasonal waves, primarily in infants and young children. This study aims to quantify the number of RSV‐related hospitalizations in children ≤2 years of age and to determine corresponding resource use and costs in Germany.

**Methods:**

We retrospectively analyzed population‐wide hospital data from the Institute for the Hospital Remuneration System (InEK) from 2019 to 2022. RSV cases were identified using the RSV‐specific 10th revision of the International Classification of Diseases (ICD‐10) codes J12.1, J20.5, and J21.0. The RSV‐associated proportion of all hospitalizations caused by severe acute respiratory infections (SARIs), clinical manifestations, length of stay (LOS), intensive care unit (ICU) admissions, ventilation rates, and hospitalization costs were retrieved.

**Results:**

We identified 98,220 hospitalizations (26,052, 15,407, 31,362, and 25,399 in 2019, 2020, 2021, and 2022, respectively) with a principal RSV diagnosis in children aged ≤2 years in Germany. The majority of RSV hospitalizations (73,178) occurred in infants (<1 year), with annual incidence rates ranging from 14.9 to 28.6 per 1000 population. Fifty‐eight percent of all SARI hospitalizations in this age group were attributable to RSV. In children aged ≤2 years, mean LOS was 4.5 days, 6.1% of cases were admitted to ICU, and 5.3% of cases were ventilated. Mean hospitalization costs per case ranged from €3001 to €3961 over the study period.

**Conclusions:**

RSV causes substantial disease burden and is a leading cause of SARI‐related hospital admissions of children ≤2 years of age in Germany. Our results confirm the need to explore and evaluate strategies to prevent RSV in infants and young children.

## 1 BACKGROUND

Respiratory syncytial virus (RSV) is a major cause of lower respiratory tract infection (LRTI) in young children worldwide,^1^, ^2^, ^3^ with bronchiolitis being the most common clinical manifestation of RSV‐related LRTI in infants.^4^, ^5^, ^6^, ^7^, ^8^, ^9^ Compared with influenza, RSV causes up to 16 times more hospital stays and emergency room visits in children younger than 5 years.^1^ In a recently updated systematic review and meta‐analysis on childhood disease burden of RSV‐related LRTI, it was estimated that 33.0 million LRTI cases and 3.6 million LRTI hospital admissions were due to RSV in children aged 0–60 months globally in 2019.^10^ The same study estimated the incidence of RSV‐related LRTI in high‐income countries to be 38.5 per 1000 infants aged 0–12 months. Accordingly, most RSV‐related hospitalizations occur in the first year of life, with a peak in the first few months of life.^1^, ^4^, ^9^, ^10^, ^11^, ^12^, ^13^, ^14^, ^15^ A study in seven European countries showed that children born 2 months before the peak of the RSV season were more likely to be hospitalized due to RSV in the first year of life.^16^ Compared with term infants, preterm infants are at increased risk for RSV‐related LRTI and a severe course of RSV disease, including higher RSV hospitalization and intensive care unit (ICU) admission rates and longer hospital length of stay (LOS).^5^, ^17^, ^18^, ^19^ In addition to prematurity, chronic conditions, such as congenital heart disease (CHD), are associated with a more severe course of RSV infection.^20^, ^21^ However, most children hospitalized for an RSV‐related LRTI episode do not have a history of prematurity or underlying diseases.^1^, ^5^, ^11^, ^22^

Treatment options against severe RSV infections are limited and prevention remains the most promising strategy to reduce the burden of severe RSV disease.^23^ Until late 2023, passive immunization with palivizumab, a monoclonal antibody that needs to be administered monthly during the RSV season, was the only available pharmaceutical intervention to prevent severe RSV disease in at‐risk infants. Palivizumab is indicated for children being born prematurely with 35 weeks of gestational age or less, entering their first RSV season, or children <2 years of age with bronchopulmonary dysplasia (BPD) or hemodynamically significant CHD.^24^ In 2022 and 2023, new preventive measures against RSV including a long‐acting monoclonal antibody for passive immunization of infants and a vaccine for maternal immunization were authorized in the European Union and the United States.^23^, ^25^, ^26^, ^27^ These new interventions aim to prevent severe RSV disease also in infants currently outside the population eligible to receive immunization with palivizumab.

To support the introduction of technologies such as monoclonal antibodies or maternal vaccines, data on the burden of RSV are essential. Evidence on the disease burden of RSV‐associated inpatient stays and the associated resource utilization in young children in Germany is limited and often outdated. To date, there is no comprehensive nationwide analysis that covers all RSV hospitalizations in Germany. This study aims to quantify the number of RSV‐associated LRTI hospitalizations and to describe corresponding resource use and costs in children under 2 years of age from 2019 to 2022 in Germany.

## 2 METHODS

### 2.1 Data source

We retrospectively analyzed population‐wide hospital data from the German Institute for the Hospital Remuneration System (InEK). The case‐related data are compulsory to be reported to InEK by every hospital in Germany and are accessible to the public for research purposes in anonymized form via an online data access tool. Data were extracted from the so‐called *§ 21 datasets*, which include information on all cases of hospitalization as required by law, that is, sections 1 and 3b of § 21 of the German Hospital Remuneration Act (KHEntgG).^28^ The data source allows identification and analysis of cases by principal and secondary diagnoses, pre‐specified age groups, diagnosis‐related groups (DRGs), discharge reason, LOS, ICU admissions, ventilation hours, and dates of admission and discharge. The definition of ventilation in the data includes both invasive mechanical ventilation procedures and non‐invasive respiratory support, following the German coding guideline published by InEK.^29^ The hospital discharge date determines the calendar year a case is assigned to. Due to data protection measures, subgroups with fewer than three cases cannot be investigated separately.

### 2.2 Case definitions

RSV‐associated hospitalization cases were defined using the 10th revision of the International Classification of Diseases (ICD‐10), that is, the RSV‐specific codes J12.1 (RSV pneumonia), J20.5 (acute bronchitis due to RSV), and J21.0 (acute bronchiolitis due to RSV), when coded as principal diagnosis at hospital discharge. Secondary diagnoses were not included to prevent double counting and to ensure that the analysis focuses on cases that were primarily RSV associated.

International studies have already explored broader case definitions for RSV to account for underreporting of RSV with its specific ICD‐10 codes.^4^, ^30^, ^31^ Therefore, we also extracted all acute bronchiolitis diagnoses in order to quantify the RSV‐specific proportion of bronchiolitis hospital stays. We used the ICD‐10 codes J21.1 (acute bronchiolitis due to human metapneumovirus [hMPV]), J21.8 (acute bronchiolitis due to other specified organisms), and J21.9 (acute bronchiolitis, unspecified) in addition to J21.0 to determine the total number of hospitalizations associated with bronchiolitis and the distribution of bronchiolitis‐causing pathogens.

We further calculated the proportion of RSV of hospitalizations associated with any severe acute respiratory infection (SARI). SARI was defined as hospitalization cases with a principal diagnosis of any ICD‐10 code of J09–J22 (J09–J11: influenza, J12–J18: pneumonia, J20: acute bronchitis, J21: acute bronchiolitis, and J22: unspecified acute lower respiratory infection) at hospital discharge, following the approach of the Robert Koch Institute.^32^

### 2.3 Data extraction and analysis

All RSV‐, bronchiolitis‐, and SARI‐related hospitalizations from 2019 to 2022 were extracted by age group (<1 year and 1–2 years) and by calendar year. The age groups were aggregated from the pre‐defined age groups available in the database. Information on ICU admission, ventilation, mean LOS, in‐hospital mortality, and the DRG distribution was extracted for all RSV cases.

Hospitalization incidence per 1000 population was calculated using the population of the corresponding age groups on December 31 of the respective calendar year from the German Federal Statistics Office.^33^ If the combination of year and age group resulted in three or less fatal cases, we manually aggregated groups with cases from other age groups and calculated the difference to identify the exact number of in‐hospital deaths by year and age group. The weekly number of RSV‐associated hospitalizations was extracted by age group and by calendar week (CW) of hospital discharge to investigate seasonal patterns. All calculations were carried out in Microsoft Excel 365.

### 2.4 Hospitalization cost calculations

We calculated hospitalization costs per case from a third‐party payer perspective, covering the direct costs related to an inpatient stay. For each year, we extracted frequency tables of DRGs that were allocated for RSV hospitalization cases. Due to data protection measures, DRGs allocated less than three times within the respective calendar year could not be considered. DRG distributions were stratified by age groups (<1 year, 1–2 years, and total ≤2 years) and by ICU admission status (total cases, ICU‐admitted cases, and cases with normal ward stay only).

To calculate the costs per hospitalization case, we used the German federal base rates of €3544.97, €3679.62, €3747.98, and €3833.07 for 2019, 2020, 2021, and 2022, respectively.^34^ For each DRG allocated to RSV cases in 2019, the federal base rate was multiplied with the relative DRG weight.^35^ The weighted mean and standard deviation of all allocated DRG revenues were calculated to estimate the average costs of RSV hospitalizations. After a legal change in the German DRG system coming into effect from 2020, nursing care was phased out of the fixed‐rate system. Therefore, RSV hospitalization costs from 2020 to 2022 were calculated as the sum of nursing care revenue (i.e., the mean LOS multiplied with the DRG‐specific relative nursing care weight and the fixed daily nursing care valuation rate) and the federal base rate multiplied with the respective relative DRG weights.^36^, ^37^ We applied the daily nursing care valuation rates of €146.55, €163.09, and €200.00 for 2020, 2021, and 2022, respectively.^38^ To calculate the total annual costs of RSV hospitalizations in Germany, we multiplied the number of cases with the mean costs per case. All costs are expressed in Euro (€) for the respective price year.

## 3 RESULTS

### 3.1 RSV‐associated hospitalizations

From 2019 to 2022, a total of 98,220 hospitalizations with a principal RSV diagnosis were identified in children ≤2 years of age (see Table 1 for details). The highest number of RSV‐associated hospital stays occurred in 2021 (*N* = 31,362), followed by 2019 (*N* = 26,052), 2022 (*N* = 25,399), and 2020 (*N* = 15,407). In each of the four years, infants <1 year of age were more affected than children aged 1–2 years, accounting for 72%–78% of all RSV hospitalizations in children aged ≤2 years. This corresponds to an infant hospitalization incidence of 26.2, 14.9, 28.6, and 25.5 per 1000 population in 2019, 2020, 2021, and 2022, respectively. In children aged 1–2 years, incidence ranged from 2.5 to 5.6 per 1000 population.

**TABLE 1:** Hospitalization incidence, health care resource use, and case fatality associated with RSV hospitalizations in children ≤2 years of age by year, Germany 2019–2022.

Year	Age group (years)	Population	RSV hospitalizations	Hospitalization incidence (*n*/1000)	% male	Mean length of stay (SD)	Cases with ICU admission (%)	Cases ventilated (%)	Deaths (CFR, %)
2019	<1	774,870	20,269	26.2	56.5	4.7 (3.2)	1067 (5.3)	774 (3.8)	1 (<0.01)
	1–2	1,596,547	5783	3.6	53.7	4.6 (3.7)	210 (3.6)	83 (1.4)	2 (0.03)
	Total ≤2	2,371,417	26,052	11.0	55.9	4.7 (3.3)	1277 (4.9)	857 (3.3)	3 (0.01)
2020	<1	769,380	11,429	14.9	56.7	4.7 (3.1)	807 (7.1)	775 (6.8)	2 (0.02)
	1–2	1,581,959	3978	2.5	53.5	4.4 (2.8)	186 (4.7)	52 (1.3)	2 (0.05)
	Total ≤2	2,351,339	15,407	6.6	55.9	4.6 (3.0)	993 (6.4)	827 (5.4)	4 (0.03)
2021	<1	791,254	22,644	28.6	56.3	4.4 (2.9)	1482 (6.5)	1572 (6.9)	6 (0.03)
	1–2	1,569,940	8718	5.6	54.2	4.2 (2.7)	352 (4.0)	80 (0.9)	1 (0.01)
	Total ≤2	2,361,194	31,362	13.3	55.7	4.3 (2.8)	1834 (5.8)	1652 (5.3)	7 (0.02)
2022	<1	737,780	18,836	25.5	57.1	4.5 (3.3)	1506 (8.0)	1747 (9.3)	4 (0.02)
	1–2	1,616,998	6563	4.1	54.8	4.4 (3.1)	335 (5.1)	103 (1.6)	6 (0.09)
	Total ≤2	2,354,778	25,399	10.8	56.5	4.5 (3.3)	1841 (7.2)	1850 (7.3)	10 (0.04)

Abbreviations: CFR, case fatality rate; ICU, intensive care unit; RSV, respiratory syncytial virus; SD, standard deviation.

**FIGURE 1:**
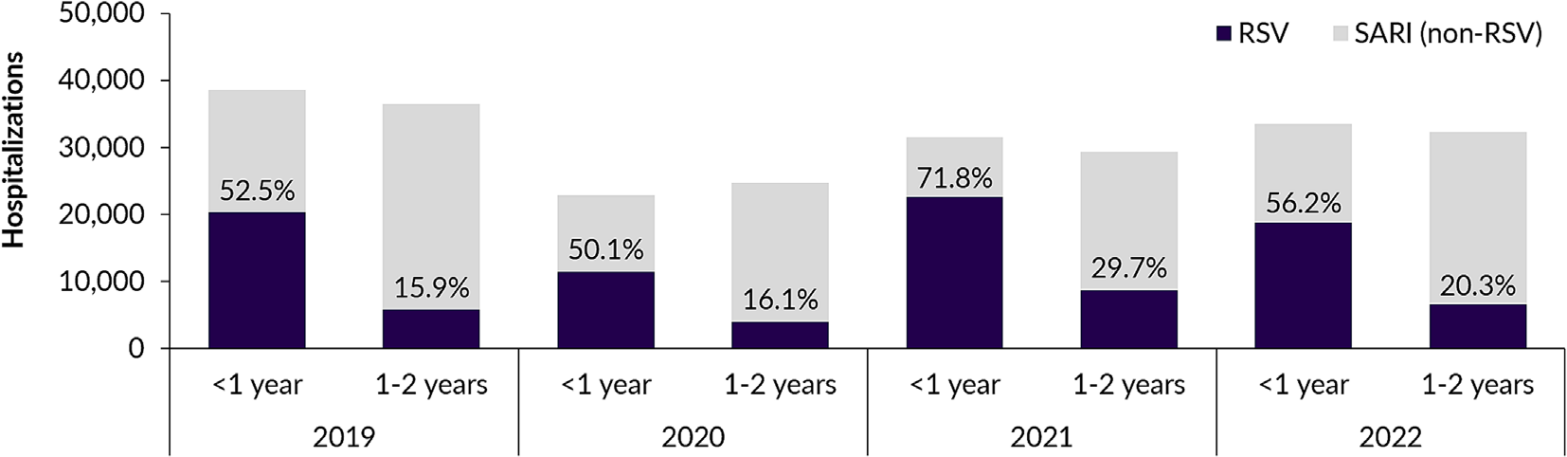
Severe acute respiratory infection (SARI) hospitalizations and proportion of respiratory syncytial virus (RSV)‐associated hospitalizations in children ≤2 years of age, by age group and year, Germany 2019–2022.

During the study period, 58% of all SARI hospitalizations in the age group of <1 year had a principal RSV diagnosis. In the age group from 1 to 2 years, this proportion decreased to 20%. The proportions of RSV among all SARI hospitalizations in children ≤2 years of age for each year are presented in Figure 1.

**FIGURE 2:**
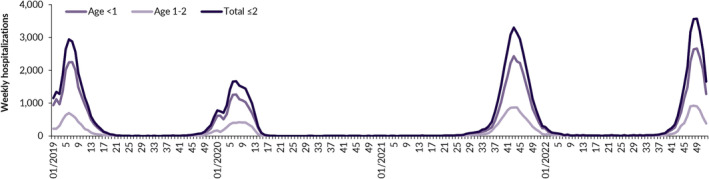
Respiratory syncytial virus‐associated hospitalizations by age group and calendar week, Germany 2019–2022.

Figure 2 shows the weekly number of RSV hospitalizations for all age groups (<1 year, 1–2 years, and total ≤2 years) over the 4‐year study period. Seasonal peaks were reached in CW6 and CW7 of the 2018–2019 and 2019–2020 season, respectively. In contrast, there was no noticeable RSV activity in 2020–2021, followed by a premature 2021–2022 season with a peak in CW43 in 2021. In late 2022, a seasonal peak was reached in CW49.

### 3.2 In‐hospital mortality

Twenty‐four deaths occurred in hospitalized children aged ≤2 years over the study period, translating into low case fatality rates between 0.01% and 0.04% from 2019 to 2022 (see Table 1). In the age group with the highest number of RSV‐associated hospitalizations (children aged <1 year), case fatality ranged from 0.005% to 0.026% over the study period.

### 3.3 Clinical manifestations

**FIGURE 3:**
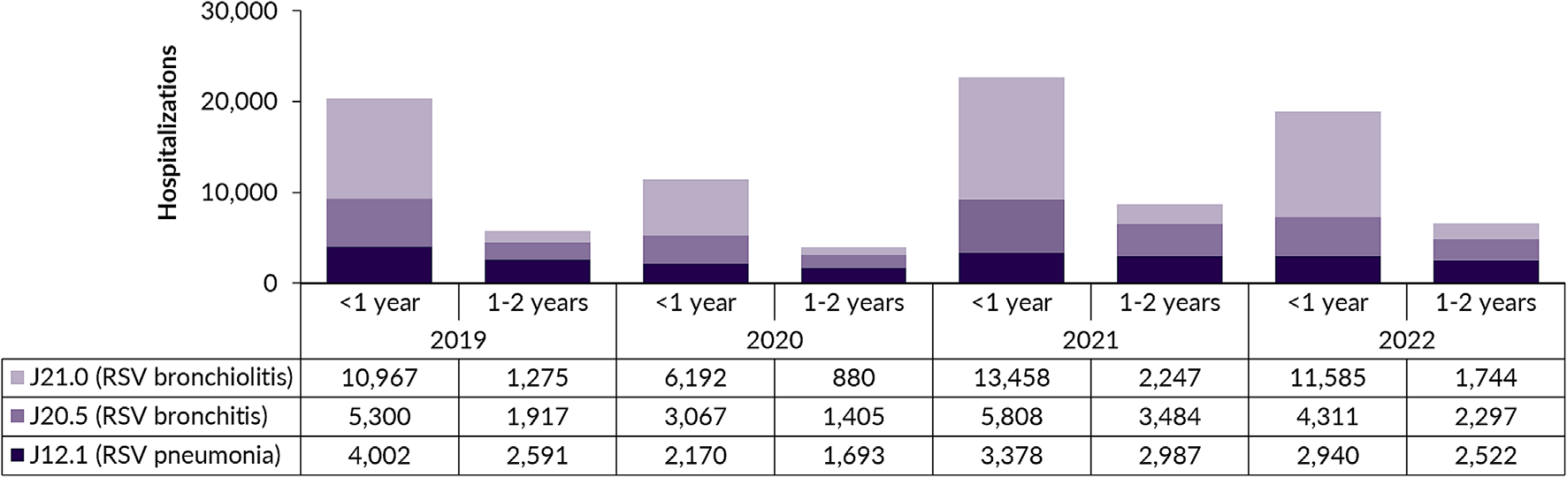
Principal diagnoses of respiratory syncytial virus (RSV) hospitalizations in children ≤2 years of age, by age group and year, Germany 2019–2022.

The distribution of RSV‐specific principal diagnoses for children ≤2 years of age for each of the four years studied is displayed in Figure 3. Bronchiolitis (J21.0) was coded as a principal diagnosis in 49% of all RSV‐associated hospital stays over the study period. Bronchitis (J20.5) and pneumonia (J12.1) contributed to 28% and 23% of all RSV hospitalizations, respectively. In children <1 year of age, bronchiolitis accounted for 58%, while bronchitis and pneumonia were responsible for 25% and 17% of all RSV‐associated cases, respectively.

Table 2 depicts the number of hospitalizations associated with bronchiolitis by principal diagnosis. Over the 4‐year study period, 91% of bronchiolitis‐associated hospitalizations of children <1 year of age were characterized with the RSV‐specific ICD‐10 code J21.0. Of the remaining bronchiolitis hospitalizations, 7% had a principal diagnosis of unspecific bronchiolitis (J21.9), and 2% were associated with J21.1 (hMPV) or J21.8 (other specified organisms). In children aged 1–2 years, 88% of hospitalizations with bronchiolitis were associated with the RSV‐specific code J21.0 over the course of 2019–2022.

**TABLE 2:** Bronchiolitis‐related hospital stays by principal diagnosis, Germany 2019–2022.

Year	Age group (years)	J21—Total bronchiolitis cases	J21.0—RSV (%)	J21.1—hMPV (%)	J21.8—Others (%)	J21.9—Unspecified (%)
2019	<1	12,332	10,967 (88.9)	46 (0.4)	229 (1.9)	1090 (8.8)
	1–2	1480	1275 (86.1)	16 (1.1)	59 (4.0)	130 (8.8)
	Total ≤2	13,812	12,242 (88.6)	62 (0.4)	288 (2.1)	1220 (8.8)
2020	<1	7052	6192 (87.8)	65 (0.9)	152 (2.2)	643 (9.1)
	1–2	1027	880 (85.7)	14 (1.4)	27 (2.6)	106 (10.3)
	Total ≤2	8079	7072 (87.5)	79 (1.0)	179 (2.2)	749 (9.3)
2021	<1	14,405	13,458 (93.4)	27 (0.2)	172 (1.2)	748 (5.2)
	1–2	2508	2247 (89.6)	12 (0.5)	63 (2.5)	186 (7.4)
	Total ≤2	16,913	15,705 (92.9)	39 (0.2)	235 (1.4)	934 (5.5)
2022	<1	12,808	11,585 (90.5)	55 (0.4)	285 (2.2)	883 (6.9)
	1–2	1953	1744 (89.3)	25 (1.3)	69 (3.5)	115 (5.9)
	Total ≤2	14,761	13,329 (90.3)	80 (0.5)	354 (2.4)	998 (6.8)

Abbreviations: hMPV, human metapneumovirus; RSV, respiratory syncytial virus.

### 3.4 Medical resource utilization and hospitalization costs

ICU admission rates varied with age and observation year (see Table 1). The ICU admission rate for under‐1‐year‐olds was 6.6% and ranged from 5.3% to 8.0% over the 4‐year study period. A lower ICU admission rate of 4.3% (range: 3.6%–5.1%) was detected for children aged 1–2 years. The ventilation rate also showed to be age dependent, with 6.7% (range: 3.8%–9.3%) of infants <1 year requiring ventilation opposed to 1.3% (0.9–1.6) in the age group of 1–2 years. The mean LOS was consistent across age groups and observation years, varying from 4.2 to 4.7 days across both age groups and all years.

RSV hospitalizations in children ≤2 years of age were associated with mean costs of €3458, €3001, €3444, and €3961 for 2019, 2020, 2021, and 2022, respectively (see Table 3 for details). These costs are based on all cases independent of ICU admission status or ventilation. Considering only cases involving ICU admission, mean costs were substantially higher (€8652, €7825, €9577, and €11,045 for the respective years 2019–2022). In total, annual expenditure due to RSV‐associated hospitalizations of children ≤2 years of age from a third‐party payer perspective amounted to €90.1 million, €46.2 million, €108.0 million, and €100.6 million in 2019, 2020, 2021, and 2022, respectively.

**TABLE 3:** Mean hospitalization costs (€) per RSV case, Germany 2019–2022.

Year	Age group (years)	All cases (SD)	ICU cases (SD)	Non‐ICU cases (SD)
2019	<1	3497.68 (3343.97)	8767.17 (9362.51)	3205.23 (2280.84)
	1–2	3295.82 (2664.54)	7860.99 (9447.74)	3120.82 (1763.02)
	Total ≤2	3458.15 (3216.32)	8652.27 (9381.52)	3187.69 (2185.87)
2020	<1	3176.21 (3239.83)	8718.07 (8063.62)	2778.12 (2075.61)
	1–2	2651.02 (1681.61)	4809.47 (5362.98)	2539.26 (1155.80)
	Total ≤2	3000.63 (2775.41)	7825.21 (7286.95)	2670.86 (1712.89)
2021	<1	3636.77 (3690.17)	10,282.61 (8494.25)	3172.54 (2484.03)
	1–2	2884.44 (1773.85)	6049.81 (6670.41)	2750.29 (969.63)
	Total ≤2	3443.50 (3389.19)	9576.73 (8435.71)	3055.20 (2182.55)
2022	<1	4194.62 (4463.87)	11,665.84 (9287.33)	3516.98 (2918.00)
	1–2	3287.13 (2362.34)	6921.88 (7405.21)	3094.56 (1477.28)
	Total ≤2	3961.19 (4046.78)	11,044.88 (9108.24)	3406.63 (2630.61)

Abbreviations: ICU, intensive care unit; RSV, respiratory syncytial virus; SD, standard deviation.

## 4 DISCUSSION

Using population‐wide routine data from all German hospitals, we identified high numbers of RSV‐associated hospitalizations from 2019 to 2022, especially in infants <1 year of age. We showed that RSV is a leading cause of SARI in this age group, as more than half (58%) of SARI‐associated hospitalizations had an RSV‐specific principal diagnosis.

A major part of our study period was affected by the COVID‐19 pandemic. This was mainly reflected in the shifted seasonality, as no noticeable season occurred during the winter of 2020–2021, and early seasonal peaks of severe RSV seasons were reached in CW43 and CW49 of the years 2021 and 2022, respectively. This disruption of RSV seasonality due to public health interventions aiming to mitigate the COVID‐19 pandemic is consistently reported in national and international scientific literature.^39^, ^40^, ^41^, ^42^, ^43^, ^44^

Over the study period, the seasonality pattern of RSV hospitalizations from our data matches well with the seasonality of RSV activity described for Germany in scientific literature. A study determining epidemic seasons in Germany using RSV positivity rates reported seasonal peaks in CW3 for the 2018–2019 season, in CW6 during the 2019–2020 season, no 2020–2021 season, and a peak in CW41 in 2021 for the early onset 2021–2022 season.^40^ The seasonal peaks in the respective years in our analysis ranged between 1 and 3 weeks later, namely, in CW6 in 2019, CW7 in 2020, and CW43 in 2021, which is likely due to mixed effects of hospitalizations occurring with few days of delay after infections and our analysis being based on hospital discharge rather than hospital admission dates. For 2022, our analysis suggests seasonality comparable with the 2021–2022 season, with a slightly higher peak in CW49 in 2022. Overall, there were less RSV‐associated hospitalizations in 2022 compared with 2021. However, the condensed appearance of RSV hospitalizations and the simultaneous occurrence of influenza circulation in late 2022 might have put severe pressure on the health care system.^39^

A study analyzing RSV hospitalizations of infants and young children in Germany in 2021 reported a proportion of 11.5% of hospitalized pediatric patients with RSV infection (age ≤ 5 years, 66% of included subjects <1 year) who received respiratory support, of which 10.2% received non‐invasive support and 1.3% were mechanically ventilated.^43^ In contrast, our analysis provided a ventilation rate of 5.3% for children ≤2 years of age in the corresponding season in 2021. The considerable difference is likely attributable to the varying definitions of “ventilation” between the studies, as the hospitalization data we investigated are based on the German DRG system, in which both mechanical ventilation and respiratory support, such as continuous positive airway pressure (CPAP) or high‐flow nasal cannula (HFNC), are considered as ventilation in infants.^29^

To our knowledge, only one previous study estimated the costs associated with RSV hospitalizations of children in Germany.^45^ The study was built on data gathered from 1999 to 2001 and reported mean direct medical costs associated with RSV hospitalization of children aged ≤2 years of €2507. Assuming that the analysis was conducted in 2004 (1 year prior to publication) and taking into account the years of inflation by adjusting this cost value with the German consumer price index from the German Federal Statistics Office,^46^ the result corresponds to €3443 at 2022 price level. This fits well to our reported range of mean hospitalization costs from €3001 to €3961 from 2019 to 2022, especially considering that the German DRG system was not implemented during the data collection period of the study by Ehlken et al., and their analysis was based on the previous German per diem payment hospital reimbursement system.

Incidence rates of hospital admissions with RSV‐related respiratory infections in six European countries (Denmark, England, Finland, Norway, the Netherlands, and Scotland) were recently estimated in a study for the years 2006–2018 using a retrospective registry and population‐based modeling approach.^13^ In the study, yearly RSV incidence varied between countries, that is, from 42.4 to 90.4 per 1000 population in the age group of 0–2 months, from 16.8 to 43.6 in the age group of 3–5 months, and from 6.7 to 20.7 in children 6–11 months of age. The granularity of age groups within the first year of life makes the direct comparison with our results difficult, but our findings generally appear consistent. In a recently updated global systematic analysis, the hospitalization incidence of RSV‐associated acute LRTI was estimated to be 15.9 per 1000 children aged 0–12 months (uncertainty range: 12.6–21.2) in 2019.^10^ Narrowing down the incidence to high‐income countries only, RSV hospitalization incidence increased to 22.0 (17.1–28.4) per 1000 children in the same age group and year. Compared with our estimated incidence of 26.2 per 1000 infants aged <1 year in 2019, the incidence in other high‐income countries appears to be similar with a tendency to be slightly lower.

An important strength of our study is its representativeness, as RSV hospitalizations were identified and analyzed in a population‐wide dataset of all German hospitals. To our knowledge, this is the first study to investigate the inpatient burden of RSV in Germany from nationwide data. A further strength is that we were able to include recent data, including one pre‐COVID‐19 pandemic year and three years fully or partially affected by the pandemic. Yet, our study has several limitations. First, we focused on ensuring good specificity when extracting RSV hospitalization cases from the database, potentially at the expense of sensitivity. We excluded cases with only secondary diagnoses of RSV‐specific ICD‐10 codes to avoid double counting and to exclude cases that were primarily hospitalized for other conditions. Therefore, we could not make use of the ICD‐10 code B97.4 (RSV as the cause of diseases classified to other chapters). We do not expect considerable underestimation of hospitalization incidence due to this approach, as we expect B97.4 to be primarily used in association with upper respiratory tract infections, which are less likely to be hospitalized. Further, we exclusively used RSV‐specific ICD‐10 codes to identify RSV hospitalizations, expecting that there was reliable routine testing for RSV in German hospitals for children aged ≤2 years. Had we taken into account the ICD‐10 code J21.9 (acute bronchiolitis, unspecified), the RSV‐associated hospitalization incidence in children aged ≤2 years would increase from 11.0 to 11.5, 6.6 to 6.9, 13.3 to 13.7, and 10.8 to 11.2 in 2019, 2020, 2021, and 2022, respectively. Previous studies have suggested to explore even wider RSV case definitions to ensure sensitivity, as the clinical practice of routine testing for RSV is associated with a high level of uncertainty and potentially underestimates RSV as the cause for hospitalization with SARI.^4^, ^30^, ^31^, ^47^ Second, using hospitalization data, we were only able to quantify the inpatient burden of RSV. To get a full picture of the burden of medically attended RSV infections in Germany, outpatient treatment data are also relevant. A previous study estimated excess visits to primary care facilities in Germany due to RSV infections in the 2018–2019 season, reporting a total of 12,400 (95% confidence interval [CI] 10,400–14,600) excess visits in the age group of 0–1 years, underlining the substantial burden of RSV also in the outpatient setting.^48^ Third, our analysis was not able to differentiate between subgroups with certain risk factors or comorbidity profiles in hospitalized children. Though most hospitalizations occur in infants and young children born at term and without any known underlying comorbidities, infants with certain risk factors, such as prematurity, CHD, or BPD, have been found to have an increased individual risk for severe RSV infections.^5^, ^17^, ^18^, ^19^ Last, by structuring the analysis by calendar years, it is possible that a calendar year includes cases of two epidemic seasons. However, as we reported weekly hospitalization cases over time for the full study period in Figure 2, the seasonality of RSV hospitalizations is captured and comparison with other studies is enabled.

## 5 CONCLUSIONS

RSV causes substantial disease burden, and our study reinforces that RSV is a leading cause of SARI‐associated hospital admissions in children ≤2 years of age in Germany. With progress in clinical development of new preventive measures which led to recent authorization of a long‐acting monoclonal antibody for passive immunization of infants and a vaccine for maternal immunization, our results confirm the need to explore and evaluate strategies to prevent RSV in infants and young children.

## AUTHOR CONTRIBUTIONS

Moritz Wick and Oliver Damm conceptualized the study. Moritz Wick, Anahita Poshtiban, and Oliver Damm performed data extraction. All authors participated in data interpretation. Moritz Wick, Anahita Poshtiban, and Oliver Damm drafted the manuscript. All authors reviewed and approved the final manuscript.

## CONFLICT OF INTEREST STATEMENT

Moritz Wick, Anahita Poshtiban, Rolf Kramer, Mathieu Bangert, and Oliver Damm are employees of Sanofi‐Aventis Deutschland GmbH and may hold shares and/or stock options in the company. Martin Wetzke received fees for consulting and/or lecture services from Novartis, Pfizer, Sanofi, and AstraZeneca. Matthias Lange declares no competing interests.

### PEER REVIEW

The peer review history for this article is available at https://www.webofscience.com/api/gateway/wos/peer-review/10.1111/irv.13211.

## ETHICS APPROVAL AND PATIENT CONSENT STATEMENT

As this work did not gather patient‐ or individual‐level data or involve any interventions, formal ethical approval was not sought, and informed consent was not applicable.

## Data Availability

The data that support the findings of this study are publicly available from InEK GmbH (Institut für das Entgeltsystem im Krankenhaus) at https://datenbrowser.inek.org.
